# Surface Treatment of Carbon Nanotubes Using Modified Tapioca Starch for Improved Force Detection Consistency in Smart Cementitious Materials

**DOI:** 10.3390/s20143985

**Published:** 2020-07-17

**Authors:** Leonard Chia, Gina Blazanin, Ying Huang, Umma Salma Rashid, Pan Lu, Senay Simsek, Achintya N. Bezbaruah

**Affiliations:** 1 Department of Civil and Environmental Engineering, North Dakota State University, Fargo, ND 58108, USA; leonard.chia@ndsu.edu (L.C.); gina.blazanin@ndsu.edu (G.B.); 2 Upper Great Plains Transportation Institute, North Dakota State University, Fargo, ND 58108, USA; pan.lu@ndsu.edu; 3 Nanoenvirology Research Group, Department of Civil and Environmental Engineering, North Dakota State University, Fargo, ND 58108, USA; umma.rashid@ndsu.edu (U.S.R.); achintya.bezbaruah@ndsu.edu (A.N.B.); 4 Wheat Quality and Carbohydrate Research Group, Department of Plant Sciences, North Dakota State University, Fargo, ND 58108, USA; senay.simsek@ndsu.edu

**Keywords:** force detection, carbon nanotubes, smart cementitious materials, dispersion effectiveness, modified tapioca starch, amphiphilic co-polymer

## Abstract

The remarkable mechanical properties and piezo-responses of carbon nanotubes (CNT) makes this group of nanomaterials an ideal candidate for use in smart cementitious materials to monitor forces and the corresponding structural health conditions of civil structures. However, the inconsistency in measurements is the major challenge of CNT-enabled smart cementitious materials to be widely applied for force detection. In this study, the modified tapioca starch co-polymer is introduced to surface treat the CNTs for a better dispersion of CNTs; thus, to reduce the inconsistency of force measurements of the CNTs modified smart cementitious materials. Cement mortar with bare (unmodified) CNTs (direct mixing method) and surfactant surface treated CNTs using sodium dodecyl benzenesulfonate (NaDDBS) were used as the control. The experimental results showed that when compared with samples made from bare CNTs, the samples made by modified tapioca starch co-polymer coated CNTs (CCNTs) showed higher dynamic load induced piezo-responses with significantly improved consistency and less hysteresis in the cementitious materials. When compared with the samples prepared with the surfactant method, the samples made by the developed CCNTs showed slightly increased force detection sensitivity with significantly improved consistency in piezo-response and only minor hysteresis, indicating enhanced dispersion effectiveness. The new CNT surface coating method can be scaled up easily to cater the potential industry needs for future wide application of smart cementitious materials.

## 1. Introduction

Cementitious materials are one of the largest commodity groups consumed worldwide and are widely used in various civil engineering infrastructures. The effective monitoring of structural health and performance and detection of damage are very important in maintaining the safety and integrity of the structures and thus avoiding loss of human life due to catastrophic failure of undetected structural damage. Performance of structures made by cementitious materials can be monitored by several nondestructive evaluation (NDE) techniques such as acoustic, ultrasonic, X-ray or eddy current inspection [1,2]. However, the use of NDE methods on a structure made with cementitious materials is labor and time-intensive and needs experienced technical manpower. The skill levels and experience of the operators and engineers significantly influence the accuracy of diagnosis and thus the reliability of the NDE techniques. Therefore, automatic (as well as remote) monitoring of structures is highly desirable for efficient and objective structural health assessment [3,4]. 

In recent years, carbon nanotubes (CNTs) have aroused great interest among researchers because of their remarkable mechanical, electrochemical, piezo-responsive and other physical properties [5,6,7]. With a small amount of CNTs added to cementitious materials, a strong piezo-response effect can be added to structures and so have been considered a promising candidate to produce a smart cementitious materials [8]. A piezo-responsive smart material changes their electrical properties due to applied mechanical stresses or because of strains developed. The materials with piezo-responsive properties such as CNTs have been used in strain gauges to measure strains on structures [9,10]. The smart cementitious materials impregnated with CNTs may be an attractive and reliable way to approach real-time automated force sensing and structural health and performance monitoring. When applying CNTs in cementitious materials, its percentage is critical for the improvement in electrical and mechanical properties [11]. Previous studies demonstrated that the optimum percentage of CNTs in cementitious materials is approximately 0.1% of CNT by mass of cement, which would increase electrical conductivity and almost double the compressive strength [12,13,14,15,16].

Researchers have reported the addition of CNTs to cementitious materials assuming that CNTs would be effectively dispersed in water by direct mixing and hence in cementitious materials [17]. However, most nanomaterials including CNTs rather agglomerate in aqueous solutions because of the strong Van der Waals attraction force present and so direct mixing of CNTs in water (and then in cement mortar) might not have resulted in their best possible uniform distribution in the cement matrix. So far, the piezo-responses of such CNT-impregnated cementitious materials under dynamic loadings are inconsistent [10]. A few researchers have found that the CNTs were better dispersed in cementitious materials at low water to cement ratio [18,19] and that is a logical inference as the Van der Waals force cannot act effectively in such a medium and would be better dispersed. For a consistent force or resulted strain measurement using smart cementitious materials, effective dispersion of CNTs plays a critical role and is a major challenge. However, the dispersion of CNTs is a complicated process because of their higher aspect ratio [20]. The Van der Waals force can be nullified or reduced if electrostatic or steric repulsive forces can be introduced on the surface of the nanomaterials. Introducing electrostatic or steric forces should be carefully done such that any foreign materials applied and procedures used to achieve that should not interfere with the hydration and processing of the cementitious materials [21,22].

To improve the CNT dispersion in water (to be used in cement mortar) for a more consistent sensing property, there are two sets of potential methods which include the physical methods and the chemical methods [23]. A common physical method uses ultrasonication to convert the electrical voltage to mechanical vibrations that help in achieving good dispersion of CNTs in water. However, this technique cannot impart a long-term dispersion stability of CNTs and the temporary dispersion of CNTs does not translate to dispersed CNTs in cementitious materials [24]. It is considered prudent to use the physical methods in combination with the chemical methods to increase the wetting of the CNTs to improve the dispersion stability. While CNTs are hydrophobic in nature, the addition of hydrophilicity to the surface is considered a good approach to increase their dispersion in aqueous solutions (e.g., water) [25,26]. 

The chemical approaches would treat the outside surfaces of CNTs which involve the uses of surfactants [21,27,28,29,30], cement admixtures [28], covalent functional groups [29] or polymers [30] to achieve good CNTs dispersion in cementitious materials for a more consistent sensing purpose. The surface treated CNTs are dispersed to water before adding them to cementitious materials. Surfactants can improve the dispersion of CNTs through electrostatic and/or steric repulsions between the surfactant molecules deposited on the CNT surface. Various surfactants have been investigated by researchers including sodium dodecyl benzene sulfonate (SDBS or NaDDBS), Triton^TM^ X-100 (TX-100), gum arabic (GA) and cetyltrimethylammonium bromide (CTAB) and polyvinylpyrrolidone (PVP) [29,30,31,32]. Among all these surfactants, SDBS/NaDDBS and PVP provided relatively good dispersions of CNTs. The dispersion effectiveness with surfactants is strongly dependent on their concentration and it is reported that the surfactant to CNT mass ratio should be 4.0−6.25 for use in cementitious materials [27,28]. However, one of drawbacks of the use of surfactants as nanomaterial dispersant is the lack of connectivity of nanomaterials (CNTs) within cementitious matrix due to blocking by surfactant molecules and that affects the electrical and piezo responsive properties of nanomaterials used [22] and results in inconsistency in detecting forces or strains.

Cement admixtures such as polycarboxylate superplasticizer were also investigated to help disperse the CNTs in cementitious materials [33,34]. However, a good dispersion of CNTs using polycarboxylate requires a high concentration of sodium lignosulfonate, which is not recommended since it delays in the setting time of Portland cement [33]. Covalent functionalization of CNTs using strong acids such as nitric and sulfuric acids have been tried [34]. The experimental results showed that the acid-treated CNT-impregnated cement mortars produced a much stronger piezo-response compared to that with mortars where surfactant treated CNTs were used [34]. However, the application of acid treatment to disperse CNTs is difficult to scale up and the process may cause safety hazards and endanger workers. Additionally, the acid-treated dispersed CNTs could not form a well-connected three-dimensional network required for good electrical conductivity to achieve piezo-responsive properties as they had fewer contact points since it has fewer covering of surface by C-S-H phases [35]. Polymers such as acrylic acid and methyl cellulose have also been studied for surface treatment of CNTs to improve their dispersion in cementitious materials [36,37,38,39]. The reported studies mostly focused on the mechanical properties of the final cementitious materials and did not specifically look into the effectiveness of these polymer modifications in improving sensing consistency of the CNT-impregnated materials [40]. 

Base on the literatures above, there are challenges of the current surface treatment methods on CNTs for wide applications in smart cementitious materials, including (a) the scalability of the surface treated CNTs in cement base for a consistent sensing; and (b) the environmental concerns for a safe wide adoption in construction industry. To meet these challenges, nature polymer which is environmentally friendly may provide a possible solution. When reviewing options of natural polymers for potential application in cementitious materials, tapioca starch extracted from the storage roots of the cassava plant had been studied as concrete additive to increase the setting time, workability and mechanical properties of the cementitious material increases with improvements [41,42]. Recently, Rashid [43] used octenyl succinic anhydride (OSA) modified tapioca starch to coat and effectively disperse iron nanomaterials in water. The use of OAS to modify starch is a recognized chemical approach to introduce the biopolymer of starch such as tapioca starch into industrial applications. With respect to the rheological properties of OSA modified starches in aqueous medium, evidence have been provided to suggest that OSA side chains can aggregate into hydrophobic domains in water at even quite low concentrations [44,45]. 

The OSA-modified tapioca starch is amphiphilic in nature, which can potentially functionalize CNTs via non-covalent wrapping [45]. The physical adsorption of these copolymers onto CNTs surfaces increases the hydrophobic feature of the OSA, which may effectively prevent the aggregation of CNTs. Moreover, the electrostatic/steric repulsive forces of the copolymer may also overcome some Van der Waals force between CNTs. However, there was no such study reported yet in the literature for investigating the use of OSA modified tapioca starch as dispersant for CNTs in smart cementitious materials. Searching for a more consistent force measurement resulted from a well dispersed CNTs by surface treatment using an environmentally friend polymer, this study investigates the effectiveness of using OSA modified tapioca starch-based copolymer to disperse CNTs in water and thus in smart cement mortar. The amphiphilic architecture of the OSA holds promise for the copolymers’ use in CNTs dispersion for use in cementitious materials. Compared to other polymers and surfactants used as dispersants, the OSA modified tapioca starch co-polymer is a bio-copolymer, which is easy to produce and environmentally friendly. In this study, the sensing properties of the CNTs-cement mortar made by the CNTs treated with the OSA modified tapioca starch co-polymer, the NaDDBS as surfactant and without treatment (bare CNTs), were tested in laboratory. The sensing properties investigated include force sensing sensitivity, hysteresis on loading and unloading and consistency in between samples from the same mixture. If the OSA modified tapioca starch co-polymer is tested to improve the force sensing effectiveness in smart cementitious materials, it will provide a potential to solve the challenges for wide applications of CNTs enabled smart cementitious materials in construction industry. 

Accordingly, the organization of the remainder of this paper is as follows—Section 2 introduces the used material and methodology for the experimental investigation on the sensing effectiveness of different CNTs dispersion methods; Section 3 describes the experimental design, the method of data collection and signal processing; Section 4 presents the experimental results and compares the experimental results from different CNTs dispersion methods and discusses the effect of CNTs percentages on force sensitivity; Section 5 concludes the work and suggests future work.

## 2. Methodology and Materials

To investigate the impact of the use of OSA modified tapioca starch to surface treat the CNTs in smart cementitious materials on the force detection, this study prepared 36 test blocks with different CNTs dispersion methods for dynamic loading tests. All the teste blocks were prepared using the same size of 2″ × 2″ × 2.″ Table 1 shows the detailed test matrix for laboratory experiments. To study the force sensitivity of CNTs enabled smart cement mortar resulted from three different dispersion methods, including the direct mixing method using bare CNTs (Group DM), currently commercially available dispersion method using surfactant surface treatment (Group SM) and the proposed surface treatment using modified tapioca starch (Group CCNT), three groups of samples were made for each dispersion methods. Each group included six samples respectively. For comparison, all the three groups were made using the same percentage of CNTs by weight of cement of 0.1%. Previous research found 0.1% CNT by weight of cement to be optimal for the desired mechanical properties and literature also showed that 0.5% CNT was very high and led to high cost and poor performance [17,18,19,20,21]. With the same size of the test sample and the same percentage of CNTs, the density of the CNTs in each test block is expected to be the same for the resulted force sensitivity but the distribution of the CNTs in test blocks of each group may vary because different CNTs dispersion methods are applied. 

For the surfactant surface treatment method (SM), (Group SM), one approved effective surfactant in the literature, NaDDBS [29,30], was used to surface treat the CNTs (SM-CNTs) for comparison. For the surface treatment method using modified tapioca starch (Group CCNTs), the influence of percentage of CNTs was investigated in this study to optimize the CCNTs dose. For 0.1% CCNTs by weight of cement (Group A), six samples were made. Three additional CCNT/cement percentages were also studied including 0.2% CCNT—Group B, 0.3% CCNT—Group C and 0.5% CCNT—Group D. Each group of the different CCNTs percentage other than 0.1%, four test blocks were prepared for each group. Moreover, three blocks were prepared with only the modified starch (no CNTs/CCNTs, MS) added to the cement and another three blocks with only cement (no CNTs/CCNTs or modified starch, PS). The following sections detail the materials and preparations of each test groups in Table 1.

### 2.1. Surface Treatment of CNTs Using OSA Modified Tapioca Starch

CNTs Used—The CNTs used in this study are multi-walled carbon nanotubes (MWNTs, SkySpring Nanomaterials, USA) with predetermined properties (Table 2). 

**Figure 1:**
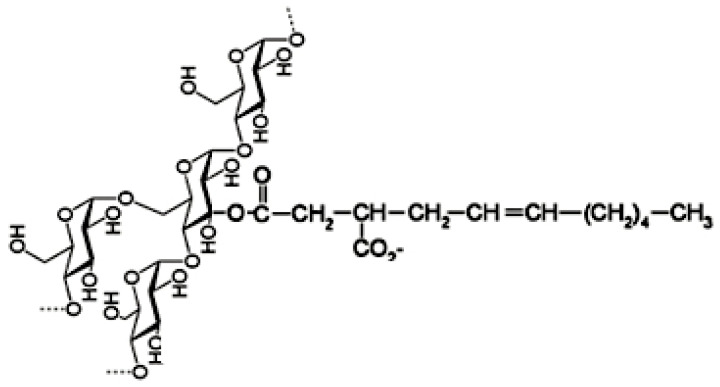
Chemical components of octenyl succinic anhydride (OSA) modified tapioca starch (drawn based on Reference [43]).

Tapioca Starch Modification using OSA–The modified tapioca starch copolymer coated CNTs (CCNTs) are expected to exhibit steric repulsions between the co-polymer molecules deposited on the surfaces of CNT surface. The OSA modified tapioca starch copolymer was prepared as per Rashid el al. (2019) [43] and others [46,47]. Native (unmodified) tapioca starch was combined with OSA to create an amphiphilic starch as shown in Figure 1 for the expected chemical structure [adopted from [41]]. More details on the co-polymer’s properties such as the FTIR spectra, the magnetic resonance and stability, refer to Reference [43]. Briefly, 100 g of tapioca starch was mixed by constant stirring in 225 mL of deionized (DI) water in a 500 mL flask while maintaining the pH of the slurry at 8.5–9.0 by controlled addition of 1M NaOH. Then 35% OSA (by the mass of starch) was slowly added to the slurry using a burette and the resulting mixture was stirred for 6 h (pH maintained at ~8.5). To prevent the starch granules from swelling, 13.6% Na_2_SO_4_ was added. On completion of the reaction, the slurry was neutralized (pH 7) with 1 M HCl. The modified starch was washed at least three times by adding DI water and centrifuging the vials (2500 rpm for 15 min) and then washing once with acetone. The modified starch was then dried (40 °C in a nitrogen environment) for 24 h and stored in plastic vials.

CCNTs Aqueous Solution (Group CCNTs)—To coat CNTs, a 10 g/L OSA-modified tapioca starch solution was prepared in DI water. Starch solution was first boiled and then cooled to 50 °C and kept heated overnight with constant stirring to produce a gelatinous starch solution. CNTs (0.2 g) were combined with 50 mL of the starch solution in multiple clear glass vials. The CNT-starch mixture was sonicated for 30 min to prevent agglomeration of the nanomaterials [26] and the vials were immediately placed in a custom-made end-over-end shaker (28 rpm) and rotated for 72 h [41] for the starch to coat onto the CNTs. Then the coated CNTs (CCNTs) were centrifuged and washed with copious amount of DI water to remove excess/unattached starch. The content from each glass vial was transferred to a separate plastic vial for ease of storage and handling. Two vials of the coated CNTs (CCNTs) (0.2 g) was directly added to fresh DI water (total water volume 240 mL) and the mixture was stirred vigorously (3 min) in a magnetic stirrer before storing in the plastic vials for future use.

### 2.2. Surface Treatment of CNTs Using Chemical Surfactant NaDDBS and Direct Mixing Method

Surfactant (NaDDBS) Treated CNTs Aqueous Solution (Group SM)—The used NaDDBS in this study was provided by Sigma-Aldrich Co., USA. A critical micelle concentration of NaDDBS in water, 1.4 × 10^−2^ mol/L, was taken as the input surfactant concentration. To prepare the solution of surfactant modified CNTs (SM) solution, 1.17 g of NaDDBS was mixed with 240 ml of water using a magnetism stirrer for 5 min. CNTs (0.4 g) were added into the aqueous solution and utilizing the ultrasonicator for 2 h to make a uniformed dispersed water-CNT-NaDDBS solution. 

Bare CNTs Aqueous Solution (Group DM)—For the control group using direct mixing method, bare (unmodified) CNTs (0.4 g) was added to 240 mL water and mixed properly using ultrasonicator for 5 min.

### 2.3. Comparison of Visual Settling Behaviour of CCNTs, Surfactant Modified CNTs and Bare CNTs

**Figure 2:**
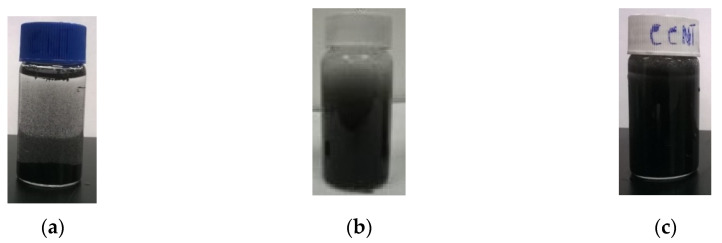
Dispersion of carbon nanotubes (CNTs) in water mixture: (**a**) Bare CNTs (Sample DM, direct mixing method); (**b**) Surface modified CNTs (Sample SM, surfactant method); (**c**) co-polymer coated CNTs (CCNTs) (Sample A, copolymer method) (Note: The inferences were made based on visual observations only. The visual observations clearly demonstrate the ability of the modified tapioca starch to disperse CNTs effectively).

The visual comparison of settling behavior of CNTs in CNT-water mixture prepared with CCNTs, surfactant modified CNTs and bare CNTs indicated that while bare CNTs settled down within 5 min (Figure 2a), surfactant modified CNTs remained partially dispersed (Figure 2b) and CCNTs remained completely suspended in water (Figure 2c). This indicated that modified tapioca starch coating may improve dispersion of CNTs in the aqueous solution. 

### 2.4. Preparation of Smart Cementitious Samples 

**Figure 3:**
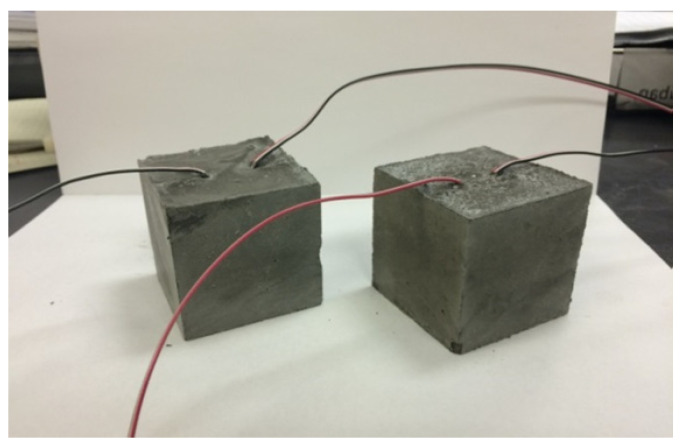
2″ × 2″ test blocks of cementitious materials. Multiple blocks were prepared with CCNT, SM and bare CNT (control) impregnated in the blocks. Additional blocks were prepared with only modified starch impregnated in the blocks and with no impregnation (bare cement). The electrical wires were fitted for the measurement of piezo-responses from the blocks when subjected to dynamic loading.

All the cement mixtures prepared in this study had a water to cement ratio of 0.6 and this ratio was maintained in all samples in this study. To prepare the CNTs-cement mixture for each group including Group CCNT, SM and DM, the CNTs aqueous solutions prepared in Section 2.1 and Section 2.2 were then added with 400 g of Portland cement (Holcim, Inc, USA), respectively. In addition, two other control groups (MS and PS) were also prepared. The control group MS prepared with only modified tapioca starch in cement mortar with 0.4 g of modified tapioca starch, 240 mL water and 400 g of cement. The other control group PS did not have any CNTs or modified tapioca starch but only 240 mL water added to 400 g of cement. The test blocks (2″ × 2″ × 2″) were prepared with the CCNT-cement, SM-cement, bare CNT-cement, modified tapioca starch-cement and plain cement mixtures. Two electrical wires were embedded in each block with their naked ends inside the block placed 2 mm apart meant for piezo-response measurement as shown in Figure 3. All the samples were prepared at room temperature (22 °C ± 2 °C) and cured for 7 days.

## 3. Experimental Set-Up and Measurement Method 

### 3.1. Experimental Setup 

**Figure 4:**
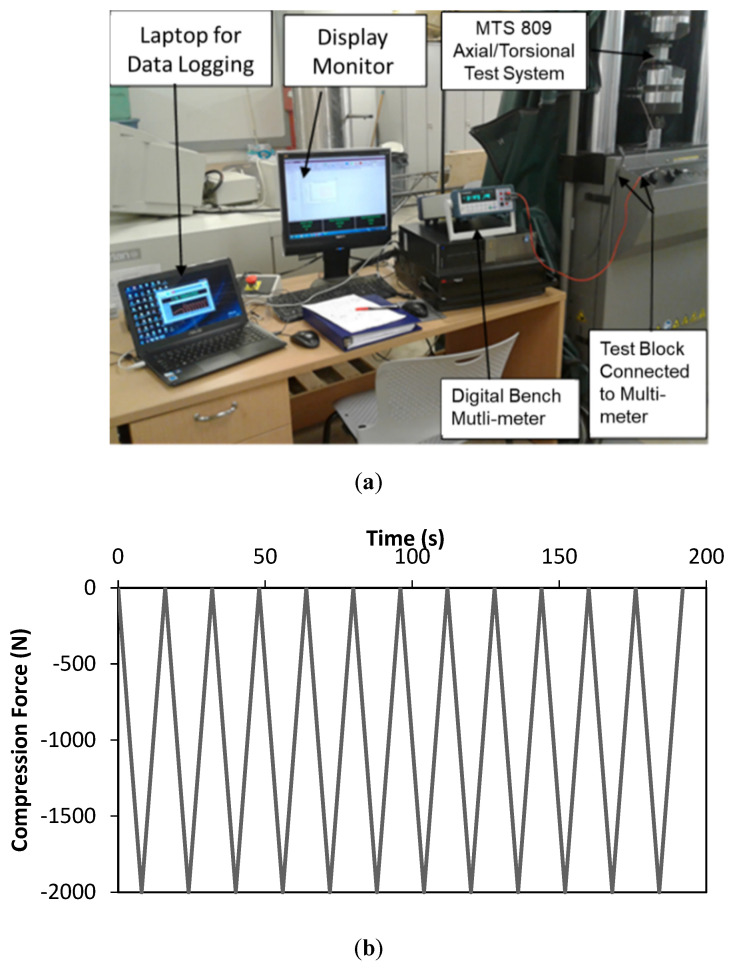
Testing of samples for piezo-responses: (**a**) Laboratory set-up for sample testing; and (**b**) 12-cycle dynamic loading scheme for the test samples.

To evaluate piezo-response characteristics of the blocks made with cementitious materials and impregnated with CCNTs, surfactant modified CNTs and bare CNTs, as well as the other two controls, dynamic loading tests were performed on the test blocks (Table 1). During dynamic load tests, the load sensing characteristics that were investigated included sensitivity, repeatability and hysteresis. An experimental set-up was designed and the instruments were interfaced with computers for display and automatic data logging (Figure 4a). Dynamic compressive loads were applied using a material testing machine (MTS 858, Materials Testing Systems, Inc., Austin, TX, USA). The dynamic load applied had an average load of 1000 N with an amplitude of 1000 N for 12 cycles (Figure 4b). The negative sign means that the applied forces on the samples are compressive forces. The frequency of the loading was set at 0.1 Hz. All the samples in Table 1 were subjected to the same loading profile as in Figure 4(b). The piezo-response changes in the CNT-impregnated cement mortar blocks were measured in the direction of the compressive stress (perpendicular to the electrodes). The electrical resistance responses were measured by a two-electrode method using a digital bench multi-meter (BK 5492B, B&K Precision, Inc., Yorba Linda, CA, USA). All the samples were tested at room temperature (22 °C ± 2 °C).

### 3.2. Definition of Loading and Unloading Sensitivity 

The piezo responses of each sample as the voltage outputs from the digital bench multi-meter (R(t) in volts), which are linearly proportional to the electrical conductivity, were recorded. To obtain loading and unloading sensitivity of the CNT-impregnated cement mortar, the recorded piezo-responses were post-analyzed by dividing the responses by the maximum load (Pmax, in kN). The dynamic force response (ΔR(t)i, the trough response) of a cement mortar block can be described as a function of piezo-response and load (Equations (1)):

ΔR(t)_i_ = R(t)_i_/P_max_ (1)

The loading sensitivity of the i loading cycle (S_L,i_) can be calculated as below (Equations (2)) where Min(ΔR(t)i) is the trough response of each cycle and Max(ΔR(t)i) is the peak response of each cycle.

S_L,I_ = │Min(ΔR(t)_i_) − Max(ΔR(t)_i_)│ (2)

The unloading sensitivity of the i loading cycle (S_U,i_) can be obtained as below (Equations (3)):

S_U,i_ = │Max(ΔR(t)_i_) − Mi(ΔR(t)_i+1_)│ (3)

## 4. Results and Discussion

### 4.1. Samples with Starch Copolymer Coated CNTs (CCNTs)

**Figure 5:**
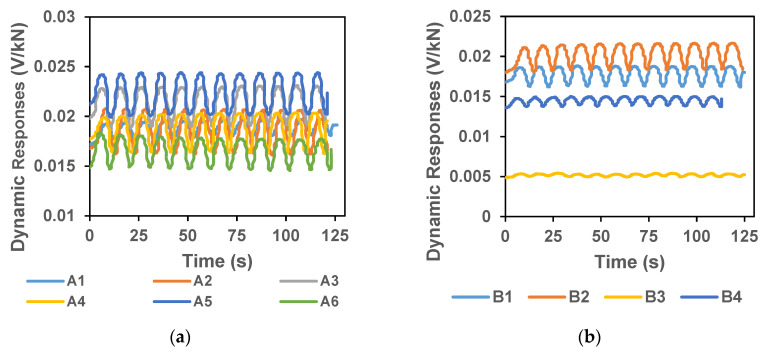

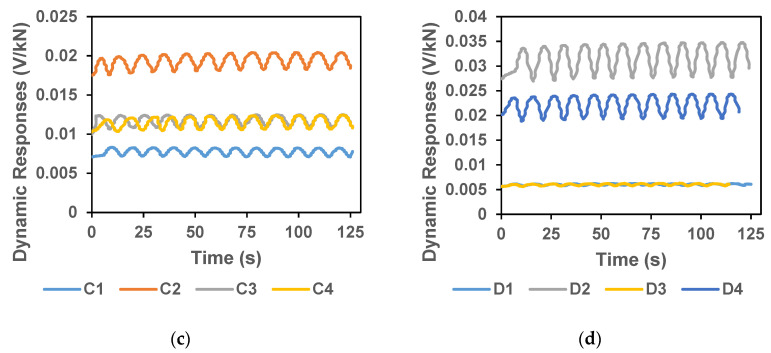
Dynamic load responses in sample blocks impregnated with 0.1% CCNTs (**a**), 0.2% CCNTs (**b**), 03% CCNTs (**c**) and 0.5% CCNTs (**d**). The multiple plots represent responses from each sample from Group B.

**Figure 6:**
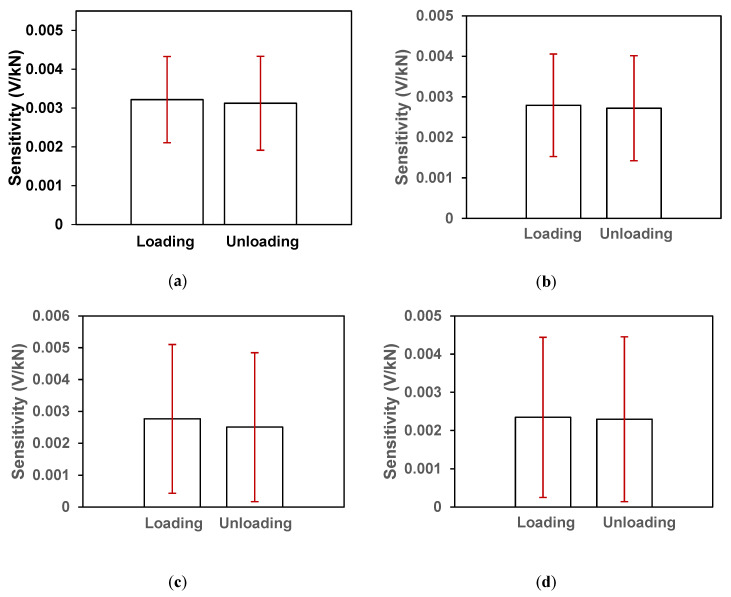
Average loading and unloading sensitivities in sample blocks impregnated with 0.1% CCNTs (**a**), 0.2% CCNTs (**b**), 0.3% CCNTs (**c**) and 0.5% CCNTs (**d**). The vertical error bars represent the standard deviations.

Figure 5a–d shows the dynamic responses (ΔR) of all the six samples prepared with 0.1% CCNTs (Group A), four samples with 0.2% CCNTs, four samples with 0.3% CNNTs and four samples with 0.5% CCNTs. It can be seen that the dynamic response of samples with 0.1% CCNTs was around 0.02 V/kN (Figure 5a). Figure 6a–d show the average loading and unloading sensitivities (S_L,i_ and S_U,i_) from the samples of 0.1%, 0.2%, 0.3% and 0.5% CCNTs and the associated standard deviations recorded. With 12-cyclic loading for each sample, there were total 72 cycles from 6 samples for 0.1% CCNTs to calculate the average values and the standard deviations. The average loading sensitivity was 0.00322 ± 0.00111 V/kN and the average unloading sensitivity was 0.00312 ± 0.00121 V/kN. For 0.2%, 0.3% and 0.5% CCNTs, 12-cyclic loading for each sample, there were total 48 cycles from four samples analyzed. In Figure 6c, d, some of the standard deviations are greater than the mean value, indicating that for 0.3% and 0.5% CCNTs, a high coefficient of variation (CV) exists in between the tested samples.

### 4.2. Samples with Surfactant Modified CNTs (SM)

**Figure 7:**
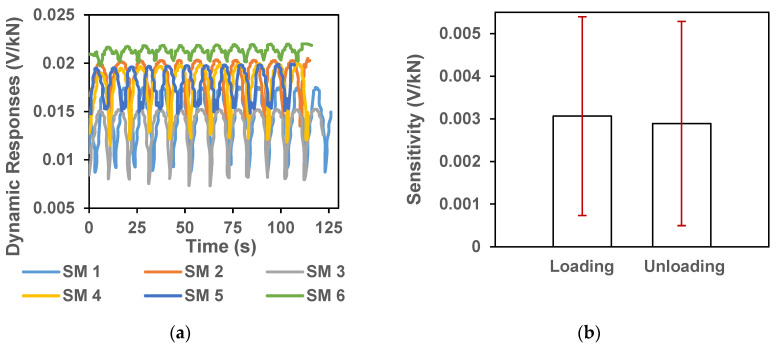
(**a**) Dynamic load responses in sample blocks impregnated with 0.1% SM-CNTs. The multiple plots represent responses from each sample from group SM; (**b**) Average loading and unloading sensitivities in sample blocks impregnated with 0.1% CCNTs. The vertical error bars represent the standard deviations.

For the six samples prepared with 0.1% surfactant modified CNTs (Group SM), the ΔR from each of the six samples were around 0.015 V/kN (Figure 7a). Figure 7b shows the average loading and unloading sensitivities (S_L,i_ and S_U,i_) and the associated standard deviations. With 12-cyclic loading for each sample, there were total 72 cycles from 6 samples to calculate the average values and standard deviations. The average loading sensitivity was 0.00307 ± 0.00233 V/kN and the unloading sensitivity was 0.00289 ± 0.00239 V/kN.

### 4.3. Samples with Bare CNTs (Direct Mixing Method)

**Figure 8:**
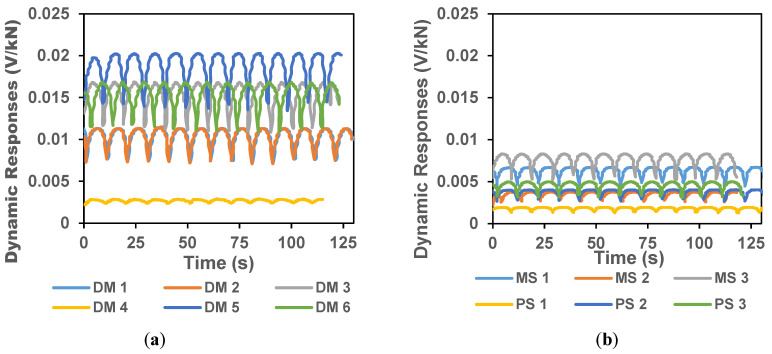
Dynamic responses in sample block impregnated with 0.1% bare CNTs using direct mixing method (**a**) and from the controls Group MS (only modified tapioca starch but no CNTs) and Group PS (no CNTs and modified tapioca starch) (**b**). The multiple plots represent responses from each sample from Group DM.

**Figure 9:**
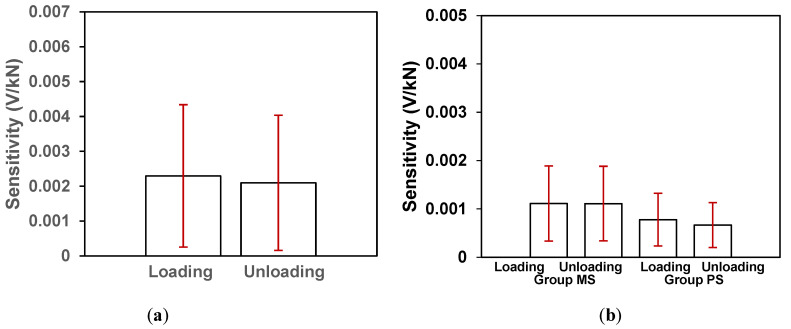
Average loading and unloading sensitivity in sample blocks impregnated with 0.1% bare CNTs using direct mixing method (**a**) and from the control Group MS (only modified tapioca starch but no CNTs) and Group PS (no CNTs and modified tapioca starch) (**b**). The vertical errors bar represents the standard deviations.

For the six samples with prepared 0.1% bare CNTs (Group DM), the ΔR from each of the 6 samples varied significantly from each other as shown in Figure 8a. The ΔR varied over large range between 0.0025 V/kN to 0.02 V/kN. The average loading and unloading sensitivities (S_L,i_ and S_U,i_) for the six samples and the associated standard deviations were recorded as shown in Figure 9a. As in the case of 0.1% CCNTs, we analyzed 72 cycles for the bare CNT blocks to obtain the average and standard deviation. The average loading sensitivity was 0.00225 ± 0.00204 V/kN and the unloading sensitivity was 0.00209 ± 0.00192 V/kN.

### 4.4. Samples with no CNTs/CCNTs (Direct Mixing Method)

Figure 8b shows the ΔR from the three sample blocks with only modified tapioca starch but no CNTs/CCNTs (Group MS) and the three sample blocks with no CNTs/CCNTs or modified tapioca starch (Group PS). The responses were either minimal or poor for samples without CNTs. The average loading and unloading sensitivities (S_L,i_ and S_U,i_) for the MS and PS samples as shown in Figure 9b were ~0.001 v/kN or lower. In the case of modified tapioca starch only (Group MS), the average loading sensitivity was 0.00111 ± 0.00078 V/KN and unloading sensitivity was 0.00121 ± 0.00077 V/kN. For Group PS (no CNTs/CCNTs or modified tapioca starch), the loading sensitivity was 0.00078 ± 0.00054 V/kN and the unloading sensitivity of 0.00067 ± 0.00046 V/kN. It can be seen that without CNTs, the piezo response of the cement mortar is insignificant. The modified tapioca starch itself slightly increases the piezo response of the modified cement mortar indicating that the modified starch might have also facilitated enhanced piezo-responses of CCNT-impregnated mortar in this study. In Figure 9a, the standard deviation of loading sensitivity is greater than the mean value, indicating that the direct mixing method introduces a high coefficient of variation (CV) in between the tested samples during loading.

### 4.5. Discussion

**Figure 10:**
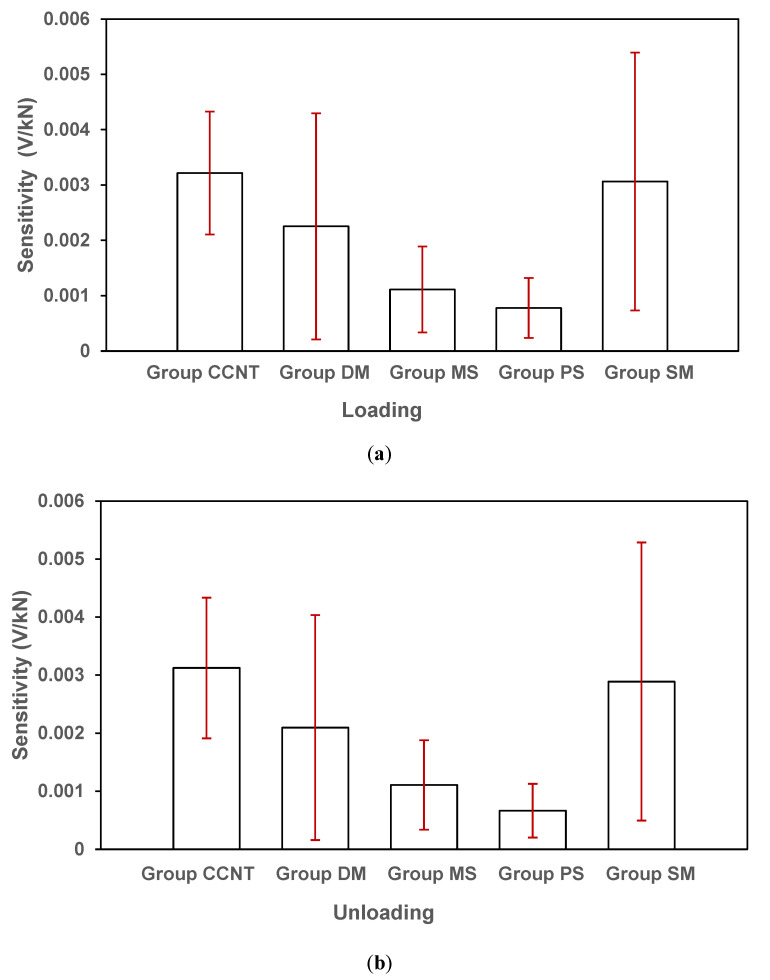
(**a**) Loading and (**b**) unloading sensitivities in the sample blocks made with CCNTs (copolymer method, Group CCNT), bare CNTs (direct mixing method, Group DM), no CNTs but copolymer (Group MS) and no CNTs and copolymer (Group PS) and surface modified CNTs (surfactant method, Group SM) and. The vertical error bars represent standard deviations.

We compared the average loading and unloading force sensitivities and hysteresis of the smart cement mortars made from with no CNTs and copolymer (Group PS), no CNTs but copolymer (Group MS), bare CNTs (direct mixing method, Group DM), chemical surface modified CNTs (surfactant method, Group SM) and CCNTs (copolymer method, Group CCNT) as shown in Figure 10a,b and Table 3. 

Force sensitivity: The samples made by surface treatment of CNTs with tapioca starch-based copolymer (CCNT) had an average force sensitivity of 0.00317 V/kN from both loading and unloading, while surfactant method had an average force sensitivity of 0.00298 V/kN and the direct mixing method was 0.00217 V/kN. The CCNT method increased average force sensitivity in the samples by ~46% compared to the samples made impregnated with bare CNTs (direct mixing method, DM). The surfactant method (SM) increased average force sensitivity in the samples by ~37% compared to the samples made impregnated with bare CNTs (direct mixing method, DM). When comparing Group CCNT with Group SM, a slight increase of average force sensitivity in the samples by ~6% was noticed. The average force sensitivity of 0.00317 V/kN was observed for the samples made with only tapioca starch-based copolymer but no CNT (MS) while the value was only 0.000725 v/kN for samples with no copolymer and CNTs. Even though the values from the control groups (MS and PS) are small, there was a 60% increase in force sensitivity due to mere use of the copolymer, so the copolymer use helped in enhancement of force sensitivity and the increase of the force sensitivity can be contributed to the nature of tapioca starch-based copolymer used for CNT surface treatment.

Hysteresis—Hysteresis describes the relation of the initial load applied to a material and its recovery rate when the load is released. The hysteresis of a material indicates how plastic or nonlinear the material is, which is significantly dependent on load time history. For a material with high hysteresis, the recovery of strain in a material subjected to a stress during its unloading cycle is incomplete due to energy consumption. A smaller hysteresis is a good indicator of a more uniform material. For a force sensing material, the less hysteresis effect it has, the better it can detect the force accurately. In this study, the average hysteresis of the smart cementitious material was evaluated by dividing the difference between the loading and unloading force sensitivities by the average force sensitivity (Table 3). For Group CCNT, the difference between the loading and unloading sensitivities is 0.0001 V/kN and the hysteresis is small with 3.1%. For Group SM, the average difference between the loading and unloading sensitivities is 0.00018 V/kN, which resulted in a hysteresis of 6.04%. For Group DM, the average difference between the loading and unloading sensitivities is 0.00016 V/kN, leading to a hysteresis of 7.4%, which is a very large hysteresis for force sensing. The tapioca starch copolymer surface treatment (Group CCNT) reduced at least half of the hysteresis compared to when bare CNTs (Group DM) and surface modified CNTs (Group SM) were used.

Repeatability/Consistency—In statistics, consistency/repeatability is a measure based on the correlations between different repetitions of measurements on the same test. The consistency/repeatability of a measurement is a good indicator for the uniformity of a sensing material. For nanomaterial (CNTs) modified smart cementitious materials, a high consistency of force measurement indicates a uniform dispersion/distribution of the nanomaterials in the cement base. Standard deviation in the data is a good measure of consistency/repeatability with a smaller standard deviation meaning a good consistency, repeatability and predictability and quality of the sensing (smart) cementitious materials. We have calculated the standard deviations of force sensitivity from all the four groups of samples in loading and unloading (Table 4). The average standard deviation was computed based on 72 measurements from Group CCNT, SM and DM. For both control groups with no CNTs (MS and PS), the standard derivations of loading and unloading were around 70% of the average force sensitivity. For with the bare CNT-impregnated cement mortar (direct mixing method, DM), the standard deviations were 90.7% for loading and 91.9% for unloading indicating a very poor consistency/repeatability in force sensing. For the surface modified CNT-impregnated cement mortar (surfactant method SM), the standard deviations were 75.9% for loading and 80.4% for unloading. For the CCNT-impregnated cement mortar, small standard deviations of 34.5% for loading and 38.8% for unloading indicated a very good consistency/repeatability for force sensing. When compared with the direct mixing method with CNTs (DM), the copolymer method increased the repeatability or consistency of the force sensing by 62.0% for loading and 57.8% for unloading. When compared with the surfactant modified method (SM), the copolymer method increased the repeatability or consistency of the force sensing by 54.5% for loading and 51.7% for unloading. 

To ensure that the comparison between sample groups is valid, a statistical analysis using Unequal Variance T-test was performed on our sample groups with confidence interval of 95% or alpha value of 0.05. The null hypothesis was that means of the two sample groups are equal. As seen from the results (Table 5), all the p-values for the comparison between the four sample groups are significantly smaller than 0.05, so the null hypothesis is rejected and a significant differences did exist between all four groups where different dispersion methods were used. 

### 4.6. Influence of the Amount of CNTs (CNT: Cement Ratio)

**Figure 11:**
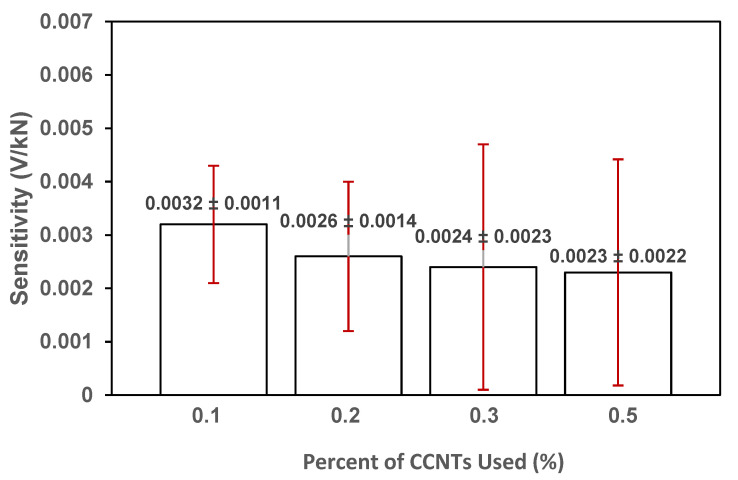
Average piezo-sensitivity in samples with different concentration of CCNTs. The vertical error bars represent the standard deviations.

For the direct mixing method, previous research had shown that 0.1% untreated CNT—Cement ratio is the optimal amount [17,18,19,20,21] for mechanical and piezo response and the increase of CNTs amount would not improve the performance. To investigate the influence of the CNT–Cement ratio in the CCNT-impregnated polymer cement mortar, comparisons were made between the average values of parameters collected from the six samples of each group (A: 0.1% CCNTs, B: 0.2%, C: 0.3% and D: 0.5%). It was observed (Figure 11) that Group A samples with 0.1% CCNTs produced the highest average loading sensitivity and showed significantly smaller standard deviation (0.0011 V/kN) compared with the other percentages of CCNTs. The results suggested that the modified tapioca starch polymer is most effective to coat the CNT surface at lower CNT concentrations. The modified tapioca starch copolymer coated CNTs (CCNTs) exhibited steric repulsions between the co-polymer molecules deposited on the surfaces of CNT surface as the CNT concentrations increase. Based on these results, we can infer that 0.1% CCNT use would be optimal. 

## 5. Conclusions

This paper investigated the improvements in measurement consistency under dynamic loading in smart cementitious materials enabled by CNTs coated with a modified tapioca starch copolymer (CCNT). The OSA modified tapioca starch copolymer with an amphiphilic architecture used for coating the CNTs helped with the effective dispersion of the CNTs in the water used for preparing the cement mortar. Based on the experimental findings, the following conclusions can be made:

(1) The CCNT-impregnated cementitious material produced higher force sensitivity with more than 40% compared to when untreated (bare) CNTs were used and a slight increase with 6% compared to when surfactant surface treated CNTs were used. 

(2) The new CCNT method significantly reduced the hysteresis of the force measurements from the resulted smart cement mortar with more than half reduction in hysteresis compared to bare CNTs and the surfactant method using NaDDBS. 

(3) The developed CCNT treatment method to CNTs introduced significant improvements in force measurement consistency for the resulted smart cement mortar, with over 62% and 50% improvement compared to the bare and surfactant treated CNTs. The improved force measurement consistency was potentially because of effective dispersion (distribution) of CCNTs in smart cementitious material. The interconnected network of dispersed CCNTs improved the electrical properties of the material. 

(4) The optimum concentration of CCNTs to be used was 0.1% by weight of cement for the best piezo-responses which complements the findings by others for optimal mechanical properties in cement mortars. With higher concentrations of CCNTs, the piezo-sensitivity was reduced. 

In this study, the developed modified tapioca starch surface treatment method was compared with the mostly common chemical surfactant method and the direct mixing method and in the future, comparisons with other effective surface treatment methods, such as the PVP surfactant method, are also recommended to further evaluate this new method. In addition, to understand the fundamentals of interaction between the CNTs and the co-polymer, detailed microscopic work (scanning electron microscopy (SEM), transmission electron microscopy (TEM)), Fourier transform infrared (FTIR) analyses, Zeta potential measurement and thermal analyses are in the progress as future work. However, with the approved enhanced force detection consistency in this study, the surface treatment method using the OSA modified tapioca starch provides a potential alternative solution to solve the challenges of the scalability and environmental concerns of the wide application of CNTs in smart cementitious materials. Thus, smart cementitious materials enabled by the OSA modified tapioca starch treated CNTs can be potentially widely used in civil structures, such as pavements, bridges, buildings and so forth, which have great needs for continuous force measurements and the resulting strain measurements; thus, to monitor the structural health and performance monitoring of the associated structures.

## Figures and Tables

**Table 1:** Test Samples Used in this Study.

Dispersion Method	Group	Number of Samples	Description
Direct Mixing Method	DM	6	0.1% CNTs
Surfactant Method	SM	6	0.1% CNTs
Copolymer Method	CCNT (A) *	6	0.1% CNTs
	CCNT (B) *	4	0.2% CNTs
	CCNT (C) *	4	0.3% CNTs
	CCNT (D) *	4	0.5% CNTs
Direct Mixing Method **	MS	3	Only Modified Starch **
Direct Mixing Method ***	PS	3	Only Cement ***

* A, B, C and D: have different CCNT amount; ** No CNTs; *** No CNTs or modified starch.

**Table 2:** Properties of the Carbon Nanotubes Used in this Study *.

Parameter	Value
Type of CNT Outside diameter	Multi−walled 50–100 nm
Inside diameter	5–10 nm
Length	5–20 um
Purity	>95 weight%
Ash content	<1.5 weight%
Specific surface area	>60 m^2^/g
Amorphous carbon content	<3.0%
Bulk density	0.28 g/cm^3^
True density	~2.1 g/cm^3^

* Supplied by the manufacturer.

**Table 3:** Comparisons of force sensitivity and hysteresis.

Group Name	Force Sensitivity: Loading (V/kN)	Force Sensitivity: Unloading (V/kN)	Sensitivity Difference (V/kN)	Average Force Sensitivity (V/kN)	Hysteresis (%)
PS	0.00078	0.00067	0.00011	0.000725	15.2
MS	0.00111	0.00121	0.00010	0.00116	8.6
DM	0.00225	0.00209	0.00016	0.00217	7.4
SM	0.00307	0.00289	0.00018	0.00298	6.04
CCNT	0.00322	0.00312	0.0001	0.00317	*3.1*

**Table 4:** Comparisons of consistency/repeatability.

Group Name	Standard Derivation: Loading (±V/kN)	Standard Derivation: Loading (%)	Standard Derivation: Unloading (±V/kN)	Standard Derivation: Unloading (%)
PS	0.00054	69.2%	0.00045	67.2%
MS	0.00078	70.3%	0.00077	63.6%
DM	0.00204	90.7%	0.00192	91.9%
SM	0.00233	75.9%	0.00240	80.4%
CCNT	0.00111	*34.5%*	0.00121	*38.8%*

**Table 5:** Statistical parameter for sample groups of different dispersion methods.

Sample Groups	t-Value	p-Value
PS * MS	−11.7509	5.16 × 10^−23^
PS * DM	−4.83611	2.14 ×10^−6^
PS * CCNT	−17.5647	9.46 × 10^−44^
PS * SM	−8.25885	5.51 × 10^−15^
MS * CCNT	12.23548	1.34 × 10^−26^
DM * CCNT	−4.31108	2.09 × 10^−5^
SM * CCNT	−9.1565	0.03 × 10^−2^
